# PubMed-Scale Chemical Concept Embeddings Reconstruct Physical Protein Interaction Networks

**DOI:** 10.3389/frma.2021.644614

**Published:** 2021-04-13

**Authors:** Blaž Škrlj, Enja Kokalj, Nada Lavrač

**Affiliations:** ^1^ Jožef Stefan International Postgraduate School, Ljubljana, Slovenia; ^2^ Jožef Stefan Institute, Ljubljana, Slovenia; ^3^ University of Nova Gorica, Vipava, Slovenia

**Keywords:** literature-based discovery, knowledge graphs, PubMed, data-mining, machine-learning, representation learning

## Abstract

PubMed is the largest resource of curated biomedical knowledge to date, entailing more than 25 million documents. Large quantities of novel literature prevent a single expert from keeping track of all potentially relevant papers, resulting in knowledge gaps. In this article, we present CHEMMESHNET, a newly developed PubMed-based network comprising more than 10,000,000 associations, constructed from expert-curated MeSH annotations of chemicals based on all currently available PubMed articles. By learning latent representations of concepts in the obtained network, we demonstrate in a proof of concept study that purely literature-based representations are sufficient for the reconstruction of a large part of the currently known network of physical, empirically determined protein–protein interactions. We demonstrate that simple linear embeddings of node pairs, when coupled with a neural network–based classifier, reliably reconstruct the existing collection of empirically confirmed protein–protein interactions. Furthermore, we demonstrate how pairs of learned representations can be used to prioritize potentially interesting novel interactions based on the common chemical context. Highly ranked interactions are qualitatively inspected in terms of potential complex formation at the structural level and represent potentially interesting new knowledge. We demonstrate that two protein–protein interactions, prioritized by structure-based approaches, also emerge as probable with regard to the trained machine-learning model.

## 1 Introduction

To this date, textual data remain one of the most widely accessible sources of information. Contemporary databases of, for example, biomedical knowledge, such as the PubMed database (Web, 2012), can consist of tens of millions of annotated scientific documents, offering, for example, detailed insights into various aspects of disease development, and the potential links between the diseases (Hristovski et al., 2005; Venkatasubbaiah et al., 2020). Albeit most of such knowledge can be accessed, it is not necessarily directly useful to a researcher, given that manual inspection of thousands of articles is not the most optimal way of uncovering, for example, how two fields of molecular biology interlink or what are the potentially interesting novel biomarkers related to a given pair of domains.

To remedy this shortcoming, the field of literature-based discovery (LBD) emerged (Swanson, 1990; Smalheiser and Swanson, 1994), exploring how contemporary computational methods can be exploited for faster and more efficient generation of potentially interesting associations, bridging different, albeit well-established concepts. Most recent LBD approaches benefit from word embeddings (Mikolov et al., 2013). A study by Tshitoyan et al. (2019) showed that latent knowledge regarding future discoveries is to a large extent embedded in past publications by retrieving information from the scientific literature with the usage of word2vec embeddings (Mikolov et al., 2013). The recent approach by Lavrač et al. (2020) explored how *word embeddings* (Joulin et al., 2016) can be used for identification of novel bridging terms in the field of plant biology. A similar approach was also explored in the context of COVID-19–related biomarker discovery (Martinc et al., 2020). An approach by Crichton et al. (2020) proposed graph-based neural network methods to perform open and closed LBD and demonstrated improved performance on existing tasks.

The purpose of this work is multifold, and its contributions can be summarized as follows.

(1) We propose CHEMMESHNET, a network of paper annotations (MeSH terms of chemicals) constructed from more than 30 million PubMed documents.
(2) The annotations, present in CHEMMESHNET, were embedded in a low-dimensional vector space via network node representation learning, which enables their direct use in downstream tasks such as discovery of *novel associations.*
(3) We demonstrate that protein representations, obtained exclusively based on document annotations, can be used to reliably reconstruct a large part of the currently known human proteome (Oughtred et al., 2020).
(4) The quantitative reconstruction results indicate that representations, based on the singular value decomposition (SVD) of a normalized graph Laplacian matrix can already offer sufficient expressive power, while scaling seamlessly to millions of links on an off-the-shelf hardware.
(5) The obtained protein representations are finally used to prioritize the space of potentially interesting *novel* protein–protein interactions. The top-ranked interactions are analyzed qualitatively at the level of protein structure.

The rest of this work is structured as follows. In Section 2, we present a brief outline of the related work, followed by the presentation of the proposed methodology in Section 3. The evaluation is presented in Section 4. Results are presented in Sections 5 and 6. Followed by the discussion in Section 7 and conclusions in Section 8.

## 2 Related Work

This section discusses the relevant related work, spanning the fields of literature-based discovery (LBD) and network representation learning. It also presents the PubMed database of biomedical articles as it is the key resource for LBD considered in this work.

The field of literature-based discovery (LBD) was conceptualized in the 1990s, when Swanson (1990) and Smalheiser and Swanson (1994) developed early LBD approaches (e.g., the so-called *ABC model*) to detect interesting bridging terms (*b-terms*), aimed at uncovering new cross-domain relations among previously unrelated concepts in separate domain corpora of interest, surveyed also by Bruza and Weeber (2008). Initial LBD works explored how lexical statistics can offer novel insights (Lindsay and Gordon, 1999). LBD has led to the discovery of potential treatments in several domains, including multiple sclerosis (Kostoff et al., 2008) and has been applied successfully in drug development and repurposing (Deftereos et al., 2011). The recent surveys offer extensive overviews of the promising approaches of LBD and their implications (Smalheiser, 2012, 2017; Sebastian et al., 2017; Thilakaratne et al., 2019). In terms of evaluation of LBD systems, Yetisgen-Yildiz and Pratt (2008) offer a comprehensive overview of the existing evaluation strategies, emphasizing that rigorous inspection of the discovered knowledge is a critical component of every LBD system.

The proposed CHEMMESHNET-based discovery focuses exclusively on biomedical knowledge discovery from the MeSH (Medical Subject Heading) terms network. Similarly, the work of Kastrin et al. (2016) was one of the first to explore how networks of co-occurring MeSH terms can be used for novel discovery. Their work served as the basis for the idea proposed in this article, where the MeSH term networks are analyzed via a node embedding–based methodology. Other promising approaches were developed for better understanding of cancer development (Pyysalo et al., 2019) using a tool LION LBD that enables researchers to navigate published information and supports hypothesis generation and testing.

A part of the proposed methodology relates to network representation learning, revolving around the notion of *node embedding*. In the recent years, instead of designing algorithms for direct link prediction (Kastrin et al., 2016) and similar tasks, development of a methodology which first projects individual nodes into a latent space (embedding) where one can directly measure similarity and, for example, predict links, has been actively explored. Methods such as DeepWalk (Perozzi et al., 2014), node2vec (Grover and Leskovec, 2016), and similar ones explore how random walk-based sampling schemes can offer compressed node representations. The node embedding methods are commonly black-box, that is, real-valued latent representations without any interpretability, many times obtained efficiently via closed-form expressions. Apart from node classification and link prediction, some other uses of node embeddings include, for example, community detection (Škrlj et al., 2020b). For a more detailed overview, the interested reader can refer to the work of Zhang et al. (2018).

Finally, we discuss the main source of knowledge used in this work—the PubMed database of biomedical articles^1^ (Sayers et al., 2020). The database receives more than 3 billion search queries each year and represents the central body of knowledge related to the biomedical domain. The current version used in this work comprises more than 30 million scientific publications, all annotated with MeSH terms. This part of annotation is of key focus to the proposed CHEMMESHNET, as it offers direct insight into into, for example, key compounds relevant for a given article, which we posit is a rich resource of human-annotated information that can be further exploited for literature-based discovery, albeit at the MeSH term graph level.

## 3 CHEMMESHNET: Construction of Concept Networks From PubMed

**FIGURE 1:**
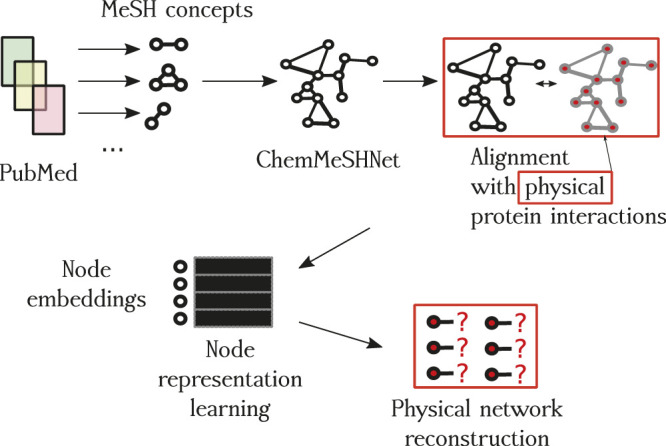
Overview of the proposed approach. The first contribution is the CHEMMESHNET, a network of term co-occurrences. Once constructed, the network is *aligned* with the space of empirically validated protein interactions (red squares), where the subset of proteins present in the annotations (nodes) of CHEMMESHNET are embedded into a low-dimensional space (node embeddings), and used to learn which links are actually present and which are not. Once trained, the best-performing classifier predicted scores for potential new interactions, which we also discuss as a part of qualitative evaluation.

**FIGURE 2:**
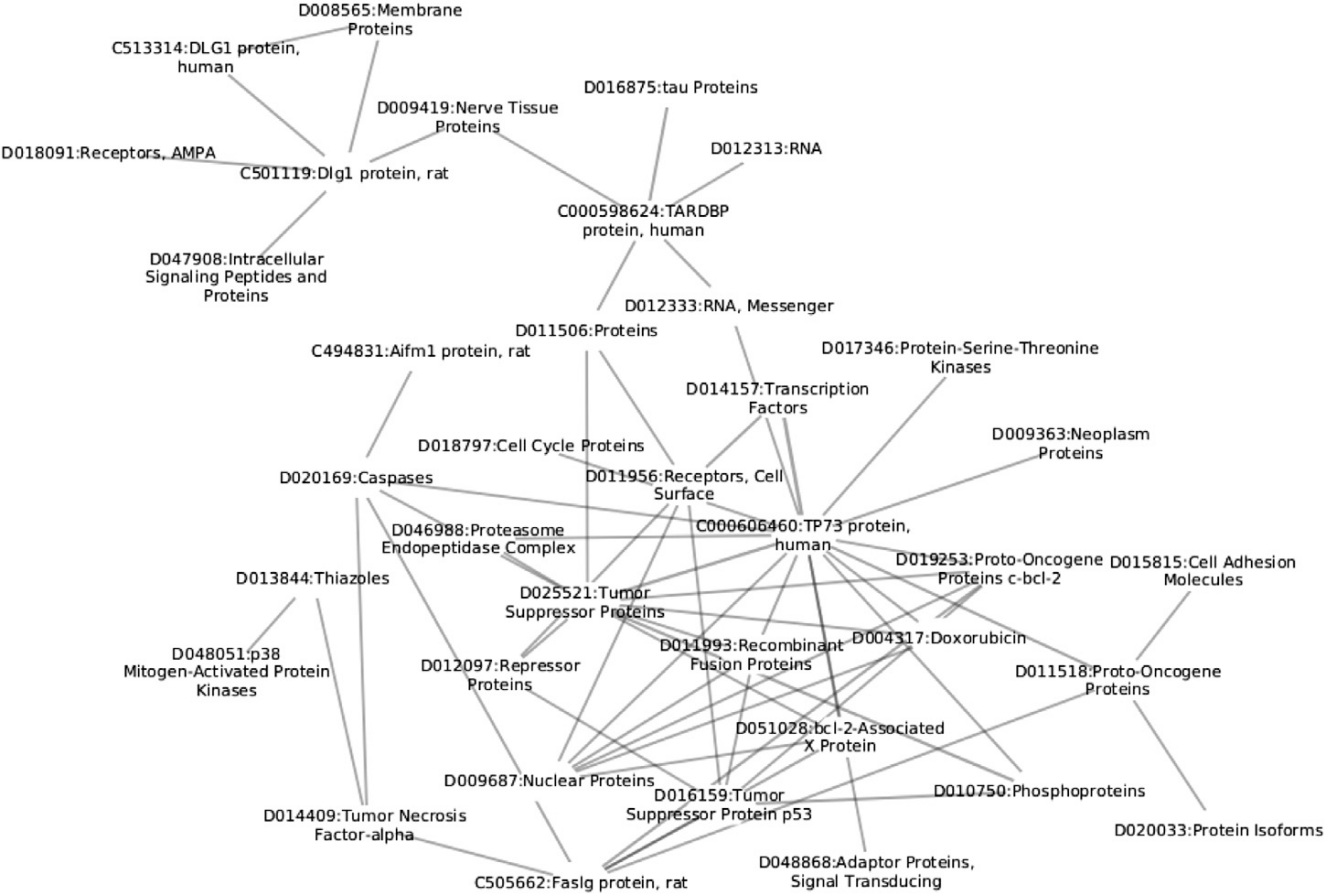
Example of MeSH chemical term subnetwork. The reader can observe different levels of information all interlinked within the same structure; from the cellular level (D018797), to protein level (C000598624).

We begin the description of the proposed methodology by first discussing the construction process of the network based on paper annotations, followed by the description of network construction and filtering. A schematic overview of the proposed approach is shown in Figure 1 and small subnetwork is shown in Figure 2.

### 3.1 Extraction of Annotations and Network Construction

In the first part of the proposed approach, we download XML representations for all available PubMed articles^2^. The XML files include abstracts and the annotations, which are the key focus of the remainder of this work. Each PubMed article has metadata, containing information on, for example, publisher, date, authors, but also the expert-curated space of *chemicals*. According to the annotators, these well-defined annotations offer insight into the *key concepts* related to the given article. All annotations of this type are also known as the MeSH tags. However, note that the considered set of MeSH tags *does not* entail all possible MeSH tags—they entail only the ones describing “chemicals”, such as proteins, compounds, and some processes. For each PubMed article (currently, there are more than 30 million articles available), we extracted these annotations and constructed a network. For example, an article with three annotations results in a triangle graph, where each of the terms is associated with all others. Intuitively, this step entails the common context. Such *cliques* are obtained for each PubMed article and joined into a single network by linking the common nodes. Further, each edge is weighted based on cumulative co-occurrence across all the documents. Schematic overview of this step is shown in Figure 1. The number of documents that have the sufficient mentioned annotation tags was 14,671,298. Note that albeit constructed from cliques, the obtained network is modular. The claim is inspected via the analysis of degree distribution in the following sections.

### 3.2 Embedding the Space of Concepts

The obtained network could be used for direct mining of the associations; however, such endeavor could be computationally expensive and prohibitive to multiple downstream tasks of interest. Hence, the nodes of the obtained network, that is, the article annotations, are then *embedded* into a low-dimensional vector space in which their semantic relations are preserved and can be directly computed.

The field of node embedding has grown in the last years; however, development of methods that scale to tens of millions of links on an off-the-shelf workstation remains an interesting research endeavor. The embedding approach employed in this work was largely inspired by the branch of methods that revolve around spectral graph decomposition, that is, the analysis of the meaning and potential implications of, for example, the largest eigenvalues of graph Laplacians and their corresponding eigenvectors (see, e.g., the work of Zhang et al. (2018)). The considered node embedding method is in one of the simplest (linear) algorithms for obtaining the representations. To embed all annotations, we implemented the node embedding as the following two-step procedure.

1. **Normalized graph Laplacian**. In the first step, we compute the normalized graph Laplacian. Let A represent a given graph’s adjacency matrix. Let D represent the degree matrix, that is, a matrix with node degrees on the diagonal and zeroes elsewhere. Next, the normalized Laplacian is computed as follows:

$$L=I-D^{-\frac{1}{2}}AD^{-\frac{1}{2}}$$

2. **Sparse Singular Value Decomposition**. In the second step, the *L* is *decomposed* into three matrices:

L≈UΣV.

The final part of this step includes re-multiplication of the first *d* diagonal entries of Σ with *U*, obtaining a low-dimensional, dense representation of individual nodes (annotations).

Let E represent the embedding (low-dimensional representation). Hence, the final result, $E\in {\mathbb{R}}^{|A|\times d}$ represents the embedding.

### 3.3 Formulating the Reconstruction Problem

The key task addressed in this work is *network reconstruction*. We formulate the task as follows. Let $G_{g}$ represent a network of *ground truth* interactions between the proteins (Oughtred et al., 2020). Commonly, the network reconstruction methods explore how node embeddings $E_{N\left(G_{g}\right)}$ can be used to reconstruct the network’s links $E\left(G_{g}\right)$. This setting operates with the representations obtained from $G_{g}$ and as such operates within the same network. However, the purpose of this study was to showcase that there exists a representation $E_{P}$, derived from PubMed, that can reliably reconstruct $E\left(G_{g}\right)$. A key part of the reconstruction of an existing network via the obtained embeddings is term alignment. We achieve this alignment via matching the protein symbol names, constrained by the *Homo sapiens* species. For example, a term that appears in CHEMMESHNET is “C102108:BACH1 protein, human.” Here, BACH1 is the protein name and human the species. The same type of identifier (and taxa) can be found in BioGrid, which we used for alignment and subsequent experiments.

To our knowledge, such endeavor is novel and was not tested before at such a scale. The main implication of being able to exploit *purely literature-based representations* of physical entities, such as proteins, is to learn potentially relevant associations between them potentially interesting discovery opportunities. We next discuss the evaluation of the proposed method that was implemented in order to be able to prioritize potential interactions between existing protein pairs from the largest currently available network of physical protein interactions.

## 4 Reconstruction Evaluation

The following section focuses on the evaluation of the proposed approach. The two main types of evaluation considered are described as follows.

The first type of evaluation focuses on the exploration of how well the network of empirically proven protein–protein interactions can be reconstructed based solely on the node (protein) representations learned from the constructed CHEMMESHNET. To quantify to what extent the relations between the proteins are learnable, we consider the task as *link prediction* and evaluate it as such. Here, we first generate a data set that captures the existing links as well as false ones, that is, pairs of protein representations that are not known to form complexes (do not interact). We obtain such node pairs by considering nodes linked with a shortest path of length, at least two. This constraint assumes that nodes that are relatively distant from one another are not expected to interact in this setting. We believe that the path-based negative sampling remains a better option to randomly selecting node pairs in terms of the amount of sampled false negatives. The constructed data set for each valid interaction considers three randomly sampled interactions at a given length that were not among the ground truth ones, that is, the negative samples.

The second type of evaluation concerns representation evaluation, which includes performance measurement of various machine-learning algorithms for the task of link prediction. The learners used in this work are the extreme gradient boosting machines (XGB) (Chen and Guestrin, 2016), random forests (RF) (Breiman, 2001), the recently introduced self-attention networks (SAN) (Škrlj et al., 2020a), Skope Rules^3^, a majority (Dummy) classifier, and a logistic regression classifier (LR). Data splitting was conducted via the *scikit-learn* library (Pedregosa et al., 2011) (Dummy and LR classifiers were implemented via this library as well). The SciPy library (Virtanen et al., 2020) was used to implement the embedding steps.

## 5 Quantitative Results Evaluation

**FIGURE 3:**
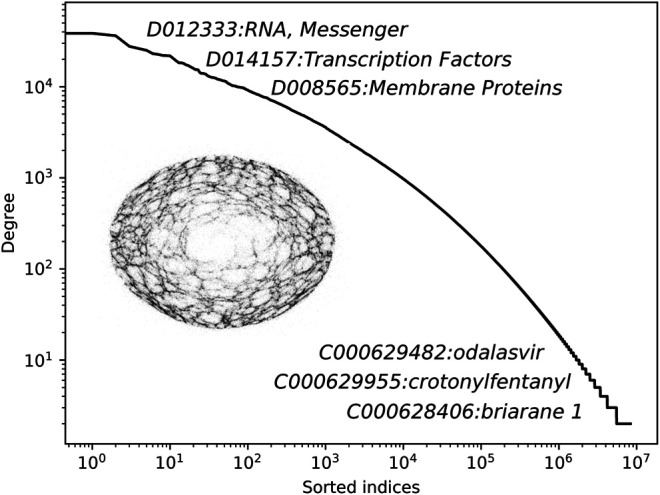
The degree distribution of the constructed CHEMMESHNET with some of the representative annotations. Note that for the purpose of this study, the constructed CHEMMESHNET consists only of MeSH terms, annotated as *chemicals*.

In this section, we present the main results of this work. We begin by describing the constructed network of annotations, followed by the quantitative reconstruction experiments. In Figure 3, we show the node degree distribution of the obtained network with annotated node names.

It can be observed that the distribution follows a linear trend in the log space, indicating that only a handful of nodes are very well connected (hubs), whereas the remaining ones are not. The distribution demonstrates that albeit clique-based construction was considered, the resulting network is far from being a regular graph. Additional statistics of the constructed network are shown in Table 1. The clustering coefficient measures how nodes in a graph tend to cluster together and is computed as the ratio between the number of closed triplets and the number of all triplets. The network density is computed as the number of actual connections, divided by all possible connections. The mean degree corresponds to the average number of connections of a node.

**TABLE 1:** Statistical overview of the constructed MeSH network of chemicals. This network is the prunned version of the one constructed directly from the MeSH annotations, as MeSH terms that are too rare: frequency <2 were not accounted for.

Property	Value
Number of nodes	54,910
Number of edges	1,308,187
Mean degree	47.64
Connected components	23
Clustering coefficient	0.724
Density	0.00087

**FIGURE 4:**
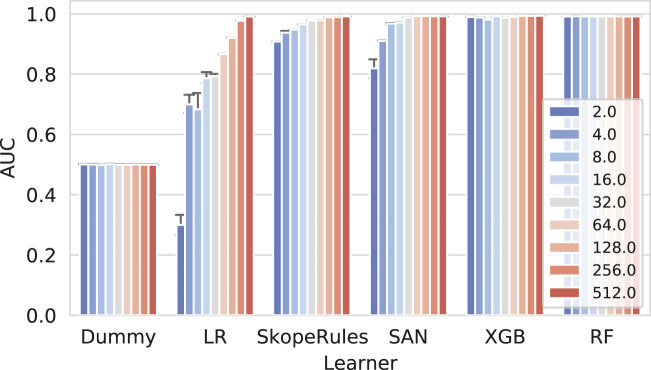
Network reconstruction benchmark results for individual learners. The more complex classifiers such as the self-attention networks (SAN), extreme gradient boosting (XGB), and Random Forests perform well when considering different amounts of training data (10, 50, and 90%). On the contrary, the logistic regression classifier (LR) performs adequately (AUC65) only if enough data were used. The impact of the embedding dimensionality can also be observed. Only high-enough dimensions result in good predictive performance, consistently for all models.

Next we present the results of the reconstruction experiment in Figure 4.

Here, a clear separation between the more complex models (neural networks and tree ensembles) and simpler ones (LR) can be observed. These results indicate that simple linear combinations of embedding dimensions are not sufficient to learn the difference between the true and false edges; however, even LR in some cases performs with AUC score ≥ 0.65, indicating that particular splits (10 splits were considered) offer differentiation even by this simpler model. As expected, the neural network–based (SAN) and tree ensemble–based (RF and XGB) models performed consistently better, with less variability. We further observed that although the neural network–based model obtained the highest overall score (AUC score 0.92), its performance was less consistent, yielding on average a negligibly worse classifier (within the deviation of the tree-based classifier). Overall, the results indicate that the reconstruction based on simple, SVD-based representations is possible, albeit only with more complex models. Note that the same data set splits were used for evaluation of all models.

## 6 Qualitative Results Evaluation

We conducted the qualitative evaluation as follows. For 1 million interactions that were not used for training and evaluation of the method, we selected the top 10,000. From these, we considered only the ones that have predicted *structural* interfaces, indicating a potentially interesting physical interaction underpinning the ML-based prediction. For each of the interactions we obtained a *score* that was the SAN’s prediction, that is, the probability of the interaction. Hence, for this step, SAN was trained on the whole positive–negative interaction sample data set and used to predict probabilities of interactions for node pairs that were not considered during training.

### 6.1 Analysis of the Predicted Interactions

The task of link prediction resulted in an extensive list of ranked interactions, which was compared to the data in two major protein–protein interaction databases that collect the information from various reliable, curated sources and are also complemented with computational predictions. The considered databases are the Interactome INSIDER and STRINGdb, from which we obtained all the interactions pertaining to human proteins. They include 112,956 and 11,759,454 annotated protein–protein interactions, respectively. After individually matching them to our ranked list, we obtained intersections of sizes 98 and 1,692, respectively. The two databases (Interactome INSIDER and STRINGdb) represent two different aspects of protein interactions. The STRINGdb, being notably larger, includes *binary* interactions, that is, interactions where only the information about whether an interaction occurs or not is an interaction known. However, such interactions are not necessarily as informative as the ones on the *structural* level. The Interactome INSIDER offers direct exploration of such interactions at the single atom resolution, potentially offering more detailed information regarding actual protein–protein binding sites. It is noteworthy that the average probability of these interactions in both cases is higher than 99%. The list of the top 55 manually curated interactions and their probabilities of the intersection between our ranked list and the Interactome INSIDER database is shown in the Appendix in Table A1. In the following subsections, we evaluate in detail two high-probability protein–protein interactions proposed by our classifier (highlighted rows in Table 3).

### 6.2 Interaction Between PTPN1 and CAPN1

The first example considers Calpain-1 catalytic subunit (UniProt: P07384) and tyrosine–protein phosphatase non-receptor type 1 (UniProt: P18031). We identified a predicted structural interface between the two proteins via the Interactome INSIDER tool (Meyer et al., 2018), which offers insight into the ECLAIR-based structural predictions^4^.

**FIGURE 5:**
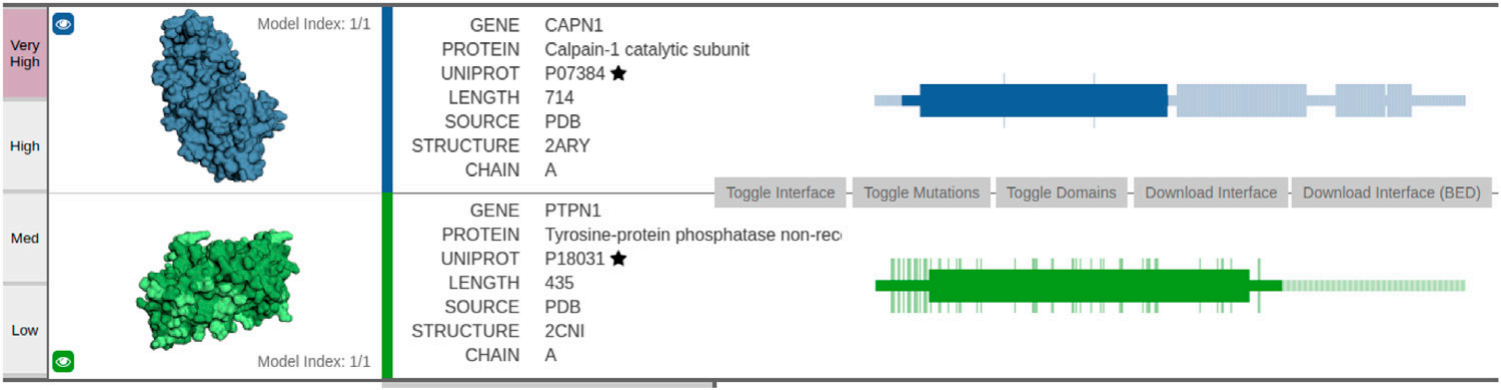
Visualization of the predicted interaction interface as obtained by the Interactome INSIDER tool. The sequential view of the amino acid sequences with annotated domain information (rectangles) is shown in the rightmost part of the figure. Note the overlap between the first domain of PTPN1 and the first domain of CAPN1—this domain overlap represents a potentially interesting protein interaction interface as predicted via ECLAIR.

The interaction interface is dark-colored in subfigures showing the structures (leftmost part of Figure 5). The first protein PTPN1—tyrosine–protein phosphatase—acts as a regulator of endoplasmic reticulum unfolded protein response. The second protein CAPN1 is a calcium-regulated non-lysosomal thiol-protease, which catalyzes limited proteolysis of substrates involved in cytoskeletal remodeling and signal transduction. The interface between the two proteins spans 468 amino acids, where numerous amino acids are predicted with very high confidence. As PTPN1 governs a signaling pathway, which modulates cell reorganization and cell–cell repulsion, the predicted interaction could offer novel insights into some of the key mechanisms of cell-to-cell signaling.

### 6.3 Interaction Between EP300 and HIF1A

The second interaction we discuss in more detail is between histone acetyltransferase p300 (UniProt: Q09472) and hypoxia-inducible factor 1-alpha (UniProt: Q16665). This association was highlighted in the task of link prediction described in Section 4. We evaluated it by retrieving the relevant information from the Interactome INSIDER tool and the STRING database of protein functional interactions (Szklarczyk et al., 2020).

**FIGURE 6:**
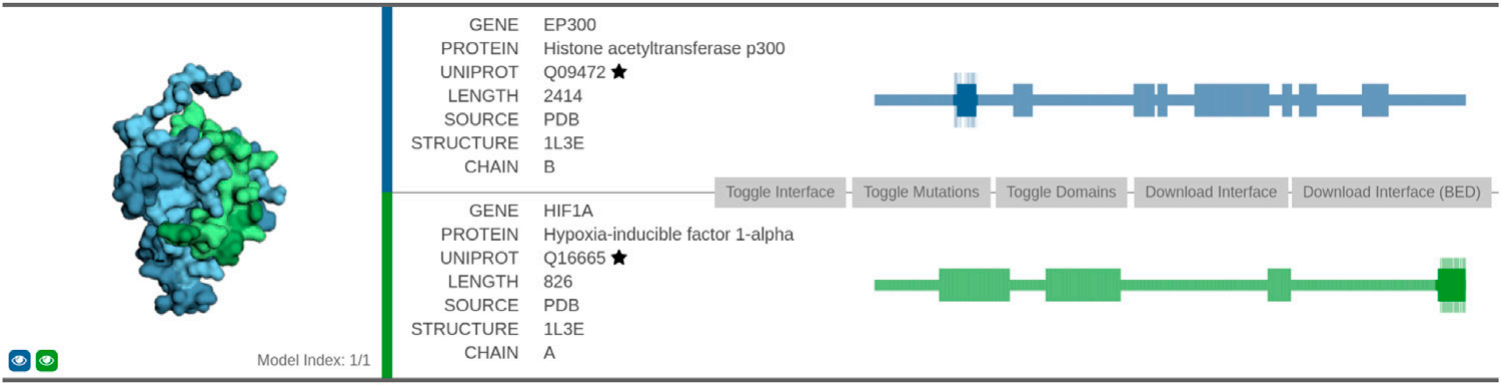
Visualization of the predicted interaction interface as obtained by the Interactome INSIDER tool. The sequential view of the amino acid sequences with the annotated domain information (rectangles) is shown in the rightmost part of the figure. The segments that form the interaction surface are shown in dark color, and the relevant amino acid residues are represented as vertical lines along the highlighted segment.

Protein EP300 functions as histone acetyltransferase and regulates transcription via chromatin remodeling, whereas protein HIF1A functions as a master transcriptional regulator of the adaptive response to hypoxia. We first analyzed the interaction using the Interactome INSIDER tool (Figure 6)^5^. The interface between the two proteins consists of 75 amino acid residues. The association of the two domains was obtained via co-crystallization experiments (leftmost part of Figure 6).

Furthermore, we also explored the confidence of the association provided by the STRING database. The STRING interaction scores represent an approximate confidence, given all the available evidence, their range is between 0 and 1. In the case of EP300 and HIF1A, the overall confidence is very high (0.998), and it is based on the following factors: the score that the interaction was experimentally determined (0.974) was obtained from an annotated database (0.900), was obtained via text-mining (0.614), and that their genes are co-expressed (0.055). The STRINGdb combines scores from separate interaction source channels by adding the probabilities together while simultaneously accounting for false discovery rate separately for each channel. Note that the interaction was not present among the ones from the BioGrid at the time of writing, indicating the proposed method’s capability to find well-represented interactions.

### 6.4 Analysis of False Positives

In the following table we present 25 selected false-positive results (prediction probability >90%). For each interaction (row) we manually inspected three existing databases for two pieces of information. First, we inspected whether a given interaction that was not present in, for example, BioGrid (stringent); could be present in STRINGdb which is much larger. And second, we identified structurally similar proteins which are known to interact, indicating that the predicted false positive is potentially a not-yet discovered interaction. The results are shown in Table 2.

**TABLE 2:** Assessment of false-positive interactions. Each of the 25 interactions was manually assessed in the three stated databases for possible presence.

Interaction	BioGRID	IntAct	STRINGdb
(‘entrez gene/locuslink:LMX1B AND entrez gene/locuslink:RP11-489N22.3’, ‘entrez gene/locuslink:FAM161A’)	✷	FAM186A	✷
(‘entrez gene/locuslink:unc-97 AND entrez gene/locuslink:F14D12.2’, ‘entrez gene/locuslink:FAM161A’)	✷	✷	✷
(‘entrez gene/locuslink:unc-97 AND entrez gene/locuslink:F14D12.2’, ‘entrez gene/locuslink:PTPN3 AND entrez gene/locuslink:RP11-18A3.3’)	✷	✷	✷
(‘entrez gene/locuslink:IGF2 AND entrez gene/locuslink:PP1446’, ‘entrez gene/locuslink:PTPN3 AND entrez gene/locuslink:RP11-18A3.3’)	✷	✷	✷
(‘entrez gene/locuslink:unc-97 AND entrez gene/locuslink:F14D12.2’, ‘entrez gene/locuslink:PXMP2’)	✷	✷	✷
(‘entrez gene/locuslink:NEU4 AND entrez gene/locuslink:LP5125’, ‘entrez gene/locuslink:PXMP2’)	✷	✷	✷
(‘entrez gene/locuslink:KIAA1958 AND entrez gene/locuslink:RP11-276E15.5’, ‘entrez gene/locuslink:NME4 AND entrez gene/locuslink:Z97634.4-011’)	✷	✷	✷
(‘entrez gene/locuslink:IGF2 AND entrez gene/locuslink:PP1446’, ‘entrez gene/locuslink:RNF40’)	RNF28, RNF29, RNF123	✷	✷
(‘entrez gene/locuslink:S100A14’, ‘entrez gene/locuslink:PXMP2’)	✷	✷	✷
(‘entrez gene/locuslink:IGF2 AND entrez gene/locuslink:PP1446’, ‘entrez gene/locuslink:FAM161A’)	✷	✷	✷
(‘entrez gene/locuslink:NEU4 AND entrez gene/locuslink:LP5125’, ‘entrez gene/locuslink:PTPN3 AND entrez gene/locuslink:RP11-18A3.3’)	✷	✷	✷
(‘entrez gene/locuslink:RPA1’, ‘entrez gene/locuslink:PXMP2’)	✷	✷	✷
(‘entrez gene/locuslink:unc-97 AND entrez gene/locuslink:F14D12.2’, ‘entrez gene/locuslink:RNF40’)	✷	✷	✷
(‘entrez gene/locuslink:KIAA1958 AND entrez gene/locuslink:RP11-276E15.5’, ‘entrez gene/locuslink:PXMP2’)	✷	✷	✷
(‘entrez gene/locuslink:LMX1B AND entrez gene/locuslink:RP11-489N22.3’, ‘entrez gene/locuslink:PXMP2’)	✷	✷	✷
(‘entrez gene/locuslink:LMX1B AND entrez gene/locuslink:RP11-489N22.3’, ‘entrez gene/locuslink:PTPN3 AND entrez gene/locuslink:RP11-18A3.3’)	✷	✷	✷
(‘entrez gene/locuslink:IGF2 AND entrez gene/locuslink:PP1446’, ‘entrez gene/locuslink:BAG5’)	BAG6	✷	✷
(‘entrez gene/locuslink:S100A14’, ‘entrez gene/locuslink:PTPN3 AND entrez gene/locuslink:RP11-18A3.3’)	✷	✷	✷
(‘entrez gene/locuslink:IGF2 AND entrez gene/locuslink:PP1446’, ‘entrez gene/locuslink:PXMP2’)	✷	✷	✷
(‘entrez gene/locuslink:KIAA1958 AND entrez gene/locuslink:RP11-276E15.5’, ‘entrez gene/locuslink:FAM161A’)	FAM124B	FAM124B	✷
(‘entrez gene/locuslink:LMX1B AND entrez gene/locuslink:RP11-489N22.3’, ‘entrez gene/locuslink:NME4 AND entrez gene/locuslink:Z97634.4-011’)	✷	✷	✷
(‘entrez gene/locuslink:unc-97 AND entrez gene/locuslink:F14D12.2’, ‘entrez gene/locuslink:LNX1 AND entrez gene/locuslink:UNQ574/PRO1136’)	✷	✷	✷
(‘entrez gene/locuslink:IGF2 AND entrez gene/locuslink:PP1446’, ‘entrez gene/locuslink:LNX1 AND entrez gene/locuslink:UNQ574/PRO1136’)	✷	✷	✷
(‘entrez gene/locuslink:unc-97 AND entrez gene/locuslink:F14D12.2’, ‘entrez gene/locuslink:BAG5’)	✷	✷	✷
(‘entrez gene/locuslink:RPA1’, ‘entrez gene/locuslink:PTPN3 AND entrez gene/locuslink:RP11-18A3.3’)	✷	✷	✷
(‘entrez gene/locuslink:KIAA1958 AND entrez gene/locuslink:RP11-276E15.5’, ‘entrez gene/locuslink:PTPN3 AND entrez gene/locuslink:RP11-18A3.3’)	✷	✷	✷

The cells marked with ‘✷’ represent no hits. If there are related binding partners, they are stated within individual cells.

**FIGURE 7:**
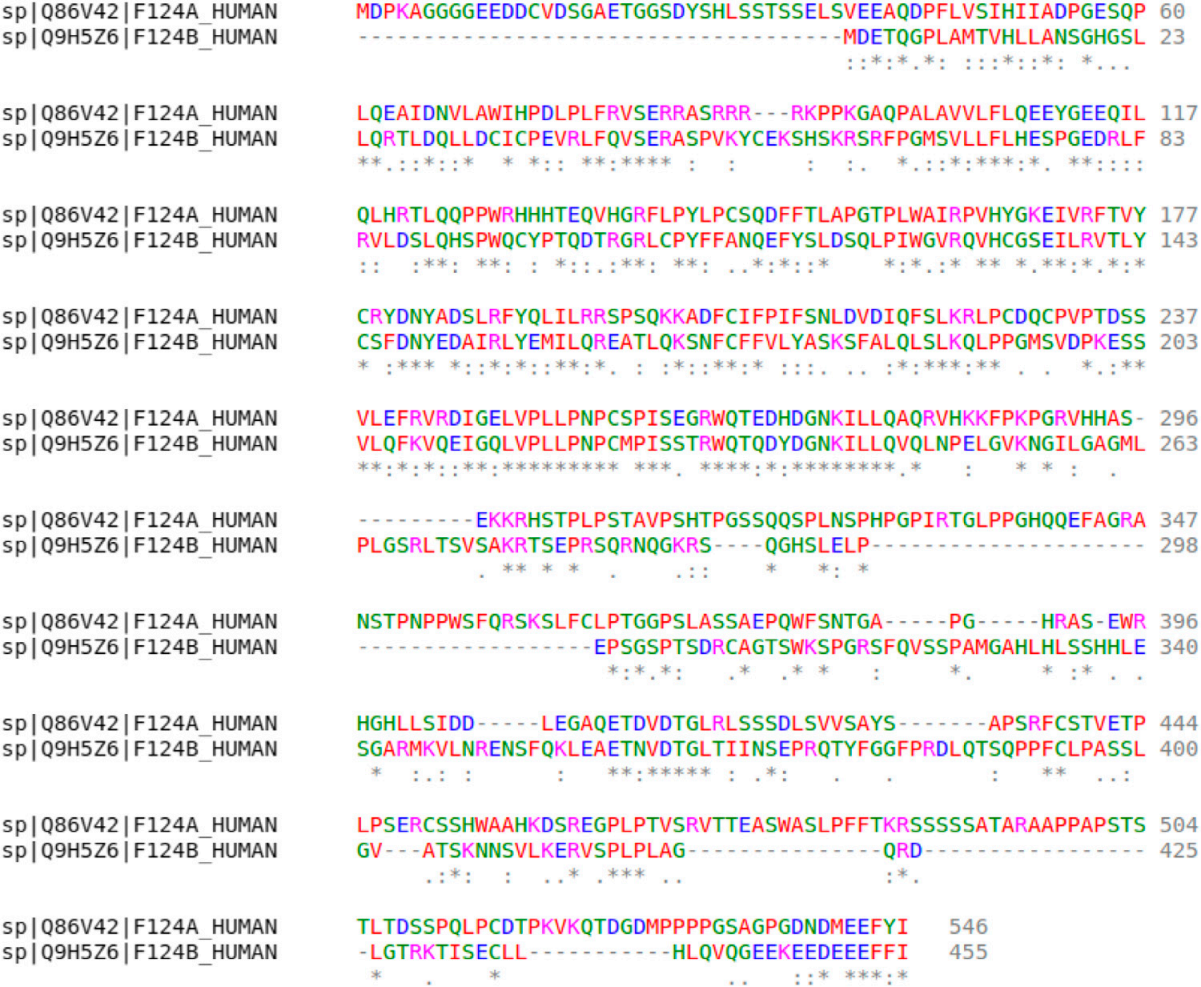
Alignment of FAM124A (present) and FAM124B identified via manual curation.

The first observation when additionally assessing the false-positive interaction is that none of the predicted interactions could be found in any of the databases via manual curation. The reasons for this result are the following. First, the high confidence criteria in STRINGdb are too stringent to unveil novel, potentially unproven interactions. Second, the manual curation could be extended to direct exploration of novel protein interaction articles; however, such manual curation was beyond the scope of this study. In terms of the observed similar interaction partners, we noticed the following: the *FAM* family of proteins (FAM124B) was identified as structurally similar to the query protein. The alignment of the two proteins is shown in Figure 7. The alignment was obtained with the Clustal Omega aligner Madeira et al. (2019).

It can be observed that parts of the two sequences overlap (approximately the residues 300–400). The overlap could indicate a similar binding site, showing a potential interaction of also FAM124B with the same protein as FAM124A. Overall, we observed that false-positive results offer an additional gateway to obtaining potentially interesting candidate interactions (apart from sampling out-of-training-distribution interactions).

## 7 Discussion

In the following section we discuss the main findings, ranging from the implications of the proposed method’s capability to reconstruct the physical networks to the discovery of novel interactions based on contextual representations of PubMed (chemicals) annotations.

The first part of this article focuses on the notion of *network reconstruction*. Here, we demonstrate that the existing BioGrid network of physical protein–protein interactions (Oughtred et al., 2020) can be adequately reconstructed by using more involved machine-learning models, such as tree ensembles or a deep neural network adapted for propositional data (SAN). We also demonstrated that simpler linear models do not perform well in this setting. The capability to reconstruct a physical network based solely on literature-based representations is a novel idea and was then extended as follows. We conducted an additional experiment, where the SAN model (one of the best-performing ones) was used to learn to estimate a *probability* of a given prediction. The model was trained on the collection of training interactions from the first experiment and used to predict probabilities for interactions, previously unseen by the model.

We qualitatively discuss such interactions, as they potentially represent *novel knowledge*. We demonstrated that one of the interactions estimated to occur (by SAN) with a high probability was in fact prioritized also by structure-only tools such as the Interactome INSIDER (Meyer et al., 2018), giving additional confidence to this interaction. We finally discuss the biological context of the interaction alongside possible implications of being able to rank millions of potentially interesting interactions. For the top prioritized interaction, we have shown that it could play a role in the cell regulation via the endoplasmic reticulum. Further, the computational predictions at the structural level obtained via Interactome INSIDER agree/confirm the predicted interaction, demonstrating the complementarity between the proposed machine learning–based and more conventional structure-based methodologies.

The focus of this work was on the relation between the literature-based MeSH tags and the existing empirical protein interaction networks. We believe that the use of biomedical named entity recognition methods, such as for example (Settles, 2005) could be additionally used for construction of knowledge graphs, extending the currently explored solution based on chemical annotations.

The considered work operates in the space of MeSH tags. There are multiple reasons for this design choice. First, memory-wise, dynamic construction of MeSH pairs is not as expensive as storing texts (possible multiple copies) during embedding construction. Second, MeSH terms are based on the *whole* article’s content, and not only the abstract. On the contrary, for the considered quantity of articles, the only sensible source of text are the abstracts (only a small percentage of the article is freely accessible). Thus, creation of embeddings based solely on abstracts could be problematic. We believe, however, that the two methodologies are compatible; both embedding-based and MeSH-based representations could be jointly used to learn potentially contextual representations which still maintain the information based on expensive manual curation.

This study focused on the chemicals-only part of the MeSH space. We believe that a natural extension of this work could include *all* MeSH tags, potentially offering richer context and applicability beyond protein/chemical networks. The current implementation of CHEMMESHNET offers direct construction of that network too; however, subsequent machine-learning experiments were beyond our computing capabilities to finish in reasonable time. The interested reader can find the instructions on how to create the extended MeSH network on the repository.

## 8 Conclusion

In this work, we presented CHEMMESHNET, a network derived from PubMed, comprising chemical-related MeSH tags. The network was hypothesized to be expressive enough to reconstruct existing physical protein–protein interaction relations, which we demonstrate quantitatively via link prediction. Further, we show how a machine-learning model, trained to recognize interactions, can be used to prioritize previously unseen interactions. We show for a pair of highly ranked interactions their overlap with the existing structure-based predictions, showcasing the added value of the proposed approach. Further, we performed extensive error analysis (manual inspection) of predicted interactions, demonstrating that this type of analysis is a potential source of novel interactions. We finally discussed the results in the context of biomedical knowledge discovery.

The proposed CHEMMESHNET serves as a freely available, mining-ready resource and is the key contribution of this article. Albeit being very expensive, we believe that it could be further extended to the space of all MeSH tags (not just chemicals). Such extensions are however potentially spatially more expensive and are therefore left for further work.

## Figures and Tables

**TABLE A1:** The top 55 protein–protein interactions and their probabilities from the intersection between our list of ranked interactions and the Interactome INSIDER database. The two marked interactions (10th and 55th) are discussed in detail in Sections 6.2 and 6.3. The ‘or’ statements in the protein columns separate different names (synonyms) for the same protein.

	Interaction probability	protein A	protein B
1	0.99999990	CAPN1 or PIG30	NIT1 or YIL164C
2	0.99999990	CAPN1 or PIG30	ATG5 or YPL149W
3	0.99999990	CAPN1 or PIG30	TINAGL1 or PP6614
4	0.99999976	CAPN1 or PIG30	LAMTOR1 or PP7157
5	0.99999940	CAPN1 or PIG30	FANCG or RCJMB04_33e6
6	0.99999810	CAPN1 or PIG30	SYNE1 or RP1-130E4.2
7	0.99999810	CAPN1 or PIG30	PTGDS or RP11-229P13.6
8	0.99999810	CAPN1 or PIG30	CASP7 or RP11-211N11.6
9	0.99999810	CAPN1 or PIG30	EEF2
*10	0.99999800	CAPN1 or PIG30	PTPN1
11	0.99999800	CAPN1 or PIG30	GBP2 or YCL011C
12	0.99999800	CAPN1 or PIG30	GINS2 or CGI-122
13	0.99999800	CAPN1 or PIG30	BZW2 or HSPC028
14	0.99999800	CAPN1 or PIG30	EIF2A or CDA02
15	0.99999800	CAPN1 or PIG30	CLEC4G or UNQ431/PRO792
16	0.99999785	CAPN1 or PIG30	EIF4E or AT4G18040
17	0.99999774	CAPN1 or PIG30	HNRNPD
18	0.99999680	CAPN1 or PIG30	EIF6 or RP4-614O4.1
19	0.99999547	EP300 or RP1-85F18.1	TINAGL1 or PP6614
20	0.99999547	TFAP2A or RP1-290I10.1	RPL15 or TCBAP0781
21	0.99999370	EP300 or RP1-85F18.1	SUMO2 or AT5G55160
22	0.99999140	TFAP2A or RP1-290I10.1	DBF4B or UNQ3002
23	0.99999130	EP300 or RP1-85F18.1	MYBL2 or AT1G71030
24	0.99999106	EP300 or RP1-85F18.1	YWHAZ or BOS_14050
25	0.99999106	EP300 or RP1-85F18.1	MAPK1
26	0.99999094	TFAP2A or RP1-290I10.1	EYA2 or RP5-890O15.2
27	0.99999080	EP300 or RP1-85F18.1	MAP3K5 or RP3-325F22.4
28	0.99999080	EP300 or RP1-85F18.1	TAF1B or Dmel_CG6241
29	0.99999070	EP300 or RP1-85F18.1	DNA2 or YHR164C
30	0.99999070	EP300 or RP1-85F18.1	PCNA or Dmel_CG9193
31	0.99999070	EP300 or RP1-85F18.1	BCAS2 or Dmel_CG4980
32	0.99999070	EP300 or RP1-85F18.1	XRCC6 or CTA-216E10.7
33	0.99999070	EP300 or RP1-85F18.1	MAGED1 or PRO2292
34	0.99999070	TFAP2A or RP1-290I10.1	FBLN1 or CTA-941F9.7
35	0.99999070	TFAP2A or RP1-290I10.1	MOB2 or C1_00620W_A
36	0.99999070	TFAP2A or RP1-290I10.1	ACTA2 or GIG46
37	0.99999070	TFAP2A or RP1-290I10.1	KCTD1
38	0.99999070	EP300 or RP1-85F18.1	YY1 or RCJMB04_1i20
39	0.99999070	EP300 or RP1-85F18.1	BCL6
40	0.99999070	EP300 or RP1-85F18.1	ENO1 or YGR254W
41	0.99999070	EP300 or RP1-85F18.1	HDAC6 or Dmel_CG6170
42	0.99999070	EP300 or RP1-85F18.1	DECR2 or AL023881.1
43	0.99999070	EP300 or RP1-85F18.1	CASK or Dmel_CG6703
44	0.99999070	EP300 or RP1-85F18.1	UBC or BOS_16579
45	0.99999070	EP300 or RP1-85F18.1	AR
46	0.99999070	EP300 or RP1-85F18.1	MYC
47	0.99999070	EP300 or RP1-85F18.1	HDAC1
48	0.99999070	EP300 or RP1-85F18.1	CRX or BOS_17597
49	0.99999070	EP300 or RP1-85F18.1	CD2 or RP4-655N15.2
50	0.99999060	EP300 or RP1-85F18.1	PLG or RP1-81D8.1
51	0.99999060	EP300 or RP1-85F18.1	TSNAX or RP11-17H4.1
52	0.99999060	EP300 or RP1-85F18.1	IGBP1 or RP13-46G5.1
53	0.99999060	EP300 or RP1-85F18.1	GOLGA2 or RP11-395P17.5
54	0.99999060	TFAP2A or RP1-290I10.1	CFH or RP1-177P10.1
*55	0.99999060	EP300 or RP1-85F18.1	HIF1A

## Data Availability

The data sets presented in this study can be found in online repositories. The names of the repository/repositories and accession number(s) can be found in the article/Supplementary Material. The code and the constructed MeSH networks are available at: https://gitlab.com/skblaz/chemmeshnet.

## Footnotes

1
https://pubmed.ncbi.nlm.nih.gov/

2
https://pubmed.ncbi.nlm.nih.gov/

3
https://github.com/scikit-learn-contrib/skope-rules

4
http://interactomeinsider.yulab.org/PPI_pair.html?interaction=P07384_P18031&mutcode

5
http://interactomeinsider.yulab.org/PPI_pair.html?interaction=Q09472_Q16665&mutcode
